# The Major Yolk Protein Vitellogenin Interferes with the Anti-*Plasmodium* Response in the Malaria Mosquito *Anopheles gambiae*

**DOI:** 10.1371/journal.pbio.1000434

**Published:** 2010-07-20

**Authors:** Martin K. Rono, Miranda M. A. Whitten, Mustapha Oulad-Abdelghani, Elena A. Levashina, Eric Marois

**Affiliations:** 1 INSERM, U963, Strasbourg, France; 2 CNRS, IBMC, UPR9022, Strasbourg, France; 3 Université de Strasbourg, UMR 963, Strasbourg, France; 4 INSERM, U964/CNRS, UMR 7104/Université de Strasbourg, IGBMC, Illkirch, France; Stanford University, United States of America

## Abstract

When taking a blood meal on a person infected with malaria, female *Anopheles gambiae* mosquitoes, the major vector of human malaria, acquire nutrients that will activate egg development (oogenesis) in their ovaries. Simultaneously, they infect themselves with the malaria parasite. On traversing the mosquito midgut epithelium, invading *Plasmodium* ookinetes are met with a potent innate immune response predominantly controlled by mosquito blood cells. Whether the concomitant processes of mosquito reproduction and immunity affect each other remains controversial. Here, we show that proteins that deliver nutrients to maturing mosquito oocytes interfere with the antiparasitic response. Lipophorin (Lp) and vitellogenin (Vg), two nutrient transport proteins, reduce the parasite-killing efficiency of the antiparasitic factor TEP1. In the absence of either nutrient transport protein, TEP1 binding to the ookinete surface becomes more efficient. We also show that Lp is required for the normal expression of *Vg*, and for later *Plasmodium* development at the oocyst stage. Furthermore, our results uncover an inhibitory role of the Cactus/REL1/REL2 signaling cassette in the expression of *Vg*, but not of *Lp*. We reveal molecular links that connect reproduction and immunity at several levels and provide a molecular basis for a long-suspected trade-off between these two processes.

## Abstract 2

Functional gene analysis in malaria mosquitoes reveals molecules underpinning the trade-off between efficient reproduction and the antiparasitic response.

## Author Summary

Malaria annually claims the lives of almost 1 million infants and imposes a major socio-economic burden on Africa and other tropical regions. Meanwhile, the detailed biological interactions between the malaria parasite and its Anopheles mosquito vector remain largely enigmatic. What we do know is that the majority of malaria parasites are normally eliminated by the mosquito's immune response. Mosquitoes accidentally acquire an infection by sucking parasite-laden blood, but this belies the primary function of the blood in the provisioning of nutrients for egg development in the insect's ovaries. We have found that the molecular processes involved in delivering blood-acquired nutrients to maturing eggs diminish the efficiency of parasite killing by the mosquito immune system. Conversely, molecular pathways that set the immune system on its maximal capacity for parasite killing preclude the efficient development of the mosquito's eggs. Our results reveal some of the molecules that underpin this example of the trade-offs between reproduction and immunity, a concept that has long intrigued biologists.

## Introduction

Malaria is a mosquito-borne parasitic disease affecting annually an estimated 250 million people, of which close to 1 million (mostly children in sub-Saharan Africa) succumb to the disease (World Health Organization fact sheet #94, April 2010; http://www.who.int/mediacentre/factsheets/fs094/en/index.html). Several *Plasmodium* species cause malaria, the most deadly being *P. falciparum* transmitted mainly by the *Anopheles gambiae* mosquito. As mosquito females require a blood meal to produce eggs, feeding on a malaria-infected host simultaneously activates oogenesis and triggers immune responses to malaria parasites. In the midgut, ingested *Plasmodium* gametocytes differentiate within minutes into gametes. After fertilization, zygotes rapidly transform into ookinetes, i.e. motile cells that traverse the midgut epithelium between 16 and 48 h post infection (hpi). Once they reach the hemolymph-bathed basal side of the midgut, ookinetes round up and transform into oocysts, protected capsules within which asexual multiplication of the parasite takes place. Previous studies have established that the ookinete is the parasite stage most vulnerable to the mosquito immune response [1],[2]. As a consequence of this response, most mosquito species efficiently eliminate all the invading ookinetes, thereby aborting the parasite cycle [3]. In a few parasite/mosquito combinations, up to 20% of ookinetes survive and the disease can be further transmitted. A number of mosquito humoral antiparasitic proteins have been characterized (reviewed in [4]). The molecularly best characterized and phenotypically most prominent defense pathway mediating the killing of *Plasmodium berghei* in *A. gambiae* involves a thioester-containing protein (TEP1) homologous to vertebrate complement factor C3 [2],[5],[6]. Depletion of TEP1 by RNA interference (RNAi) renders mosquitoes hypersusceptible to *Plasmodium* infections, resulting in abnormally high infection levels. Two leucine-rich repeat (LRR) proteins, LRIM1 and APL1C, act as TEP1 control proteins to stabilize the mature form of TEP1 in the hemolymph [7],[8] and show the same RNAi phenotype as *TEP1* in *P. berghei* infections [9]–[12]. The depletion of either protein results in precocious deposition of TEP1 on self tissues and completely aborts its binding to the ookinetes [7]. Therefore, it appears that LRR proteins regulate maintenance of mature TEP1 in circulation; however, the factors that control TEP1 targeting to the parasite surface remain unknown.

Simultaneously to the midgut crossing by ookinetes, the physiology of the mosquito is profoundly modified by a blood meal in preparation for the laying of a clutch of eggs. Within 2 to 3 d after a blood meal, the massive ovary growth allows maturation of 50–150 oocytes, a process called vitellogenesis (reviewed in [13]). The blood meal provides the mosquito with amino acids and lipids that are transferred through midgut cells to the hemolymph and signal via the Target of Rapamycin (TOR) pathway to initiate massive synthesis of nutrient transport proteins in the mosquito fat body [14]. These transport proteins include the lipid transporter lipophorin (Lp, AGAP001826) (also known as apolipoprotein II/I or retinoic and fatty acid binding protein, RFABG/P) and vitellogenin (Vg, AGAP004203), a precursor of the yolk storage protein vitellin. Both proteins are secreted into the hemolymph and transported to the ovaries. Vg is a large phospholipoglycoprotein encoded in *A. gambiae* by a small family of nearly-identical genes. Insect Vg harbors potential sites for lipidation, glycosylation, and phosphorylation and is internalized by developing oocytes where it is proteolytically cleaved to generate vitellin, a nutrient source for the developing embryo (reviewed in [15],[16]). Lp, encoded by a single transcript and post-translationally cleaved, is composed of two subunits of 250 and 80 kDa that together scaffold a lipidic particle. Similar to vertebrate low- and high-density lipoproteins (LDL and HDL, respectively), mosquito Lp particles contain a core of fatty acids and sterols, surrounded by an outer leaflet of phospholipids [17],[18]. These particles function to deliver lipids and fatty acids to energy-consuming tissues, including rapidly growing imaginal discs in larvae, muscles, and the ovary in adult females [19]. In addition to lipids, Lp particles serve as a vehicle for morphogen proteins in the imaginal discs of *Drosophila* larvae [20]. Interestingly, human HDL has been shown to host a fraction of complement factor C3 [21] as well as trypanosome-killing protein complexes [22]. In mosquitoes, recent studies [23]–[25] have implicated Lp in both mosquito reproduction and *Plasmodium* survival. In particular, experimental depletion of Lp by RNAi inhibited oogenesis and also reduced the number of developing *Plasmodium* oocysts in the mosquito midgut [23]. This could point to a nutritional requirement for Lp in the early stages of parasite development. Indeed, Lp has recently been detected by in vitro approaches inside developing *P. gallinaceum* oocysts, suggesting that it provides parasites with a source of lipids [26]. An intriguing alternative explanation is that the increasing levels of Lp following a blood meal may negatively impact mosquito immunity against parasites. Artificially blocking the physiological rise in Lp levels would then allow the immune system to exert its full strength against the parasite.

In the mosquito fat body, two distinct pathways are required for optimal expression of proteins involved in vitellogenesis: (i) the nutrient-sensing TOR pathway and (ii) a hormonal cascade that oversees production of 20-hydroxyecdysone [14],[27],[28]. Furthermore, in *Ae. aegypti* mosquitoes infected with microbes and *Plasmodium*, the NF-κB factor REL1 positively regulates expression of Lp and its receptor [24], suggesting that the NF-κB pathway may also contribute to the regulation of oogenesis in addition to its known role in mosquito immunity [29]–[31]. However, our understanding of how oogenesis and immunity impact each other remains incomplete: on one hand depletion of Lp strongly inhibits development of *P. gallinaceum*; on the other hand over-expression of *Lp* resulting from the depletion of the REL1 inhibitor Cactus in *Ae. aegypti* is insufficient to rescue the complete block in parasite development [24].

Here, we investigated the role of the two major nutrient transport proteins Lp and Vg in mosquito antiparasitic responses using a common laboratory model of malaria transmission: *A. gambiae* mosquitoes infected with the GFP-expressing rodent parasite *P. berghei* [32]. We show that similarly to Lp, Vg depletion reduces parasite survival in mosquito tissues. Strikingly however, Lp and Vg are no longer required for parasite survival if TEP1 is depleted, suggesting that the low parasite survival phenotype associated with the *Lp*/*Vg* knockdowns requires TEP1 function. We propose that Lp and Vg exert distinct non-redundant roles in reproduction and immunity: Lp is crucial for oogenesis and is required for normal Vg expression after an infectious blood meal, whereas Vg contributes to oogenesis and negatively impacts TEP1 binding to the ookinetes. We suggest that the reported negative impact of Lp depletion on ookinete survival is indirect and is mediated by reduced levels of Vg. We further demonstrate that the NF-**κ**B factors REL1 and REL2 limit the expression of *Vg* after an infectious blood meal. These results reveal an unexpected network of interactions whereby *Plasmodium* killing in mosquitoes is potentiated by NF-**κ**B pathways at two levels: (i) activation of anti-*Plasmodium* genes and (ii) inhibition of the expression of the nutrient transport protein Vg.

## Results

### Lp and Vg Depletion Reduce Parasite Survival in a *TEP1*-Dependent Manner

**Figure 1:**
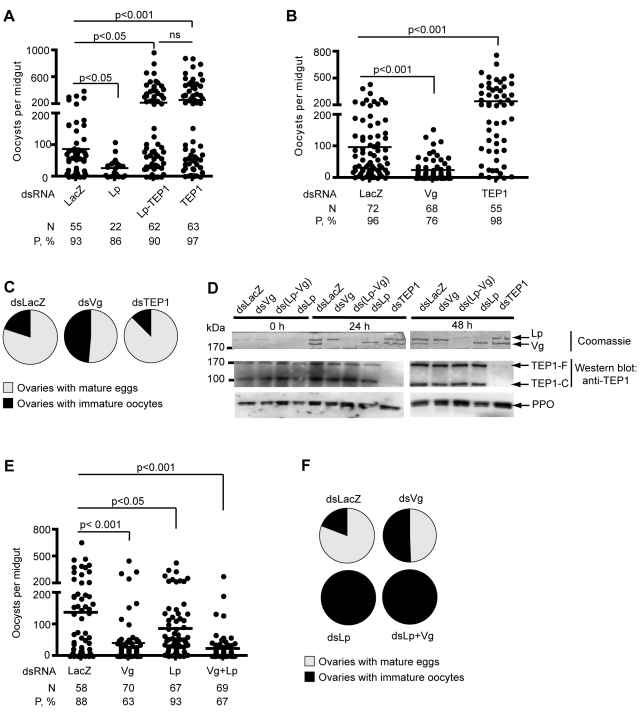
Effects of *Lp* and *Vg* silencing on parasite counts and oogenesis. Mosquitoes were injected with the indicated double-stranded RNA and infected with *P. berghei*. Parasite development was gauged 7–9 d post infection by counting GFP-expressing oocysts. Each dot represents the number of oocysts counted in one midgut. Ovaries containing mature eggs were counted 7 d post infection. Pie charts show the percentage of mosquitoes containing mature eggs (grey) versus percentage of mosquitoes containing only undeveloped oocytes (black). (A) Effect of concomitant silencing of *TEP1* and *Lp* on parasite survival. (B) Effect of *Vg* silencing on parasite survival. In (A) and (B), one representative experiment out of 4 independent replicates is shown. The additional replicates are shown in Figure S3. (C) Effect of *Vg* silencing on oogenesis. (D) Coomassie staining (top panel) of a PVDF membrane allows visualization of Vg and Lp in control, Vg-, and Lp-depleted mosquitoes. Hemolymph proteins were separated on a denaturing SDS-polyacrylamide gel and transferred to a PVDF membrane. Western blotting analysis of hemolymph of Vg- and Lp-depleted mosquitoes 0, 24, or 48 h after infection using anti-TEP1 antibody (bottom panel). (E, F) Parasite counts (E) and oogenesis (F) in Lp- and Vg-depleted mosquitoes.

*Lp* knockdown causes a decrease in parasite loads and simultaneously arrests oogenesis [23]. We examined whether the *Lp* knockdown phenotype requires the antiparasitic factor TEP1. To this end, we compared the numbers of surviving parasites in single *TEP1* or *Lp* knockdown mosquitoes and in double *TEP1/Lp* knockdowns by injecting double-stranded RNA (dsRNA) resulting in RNAi. Four days after dsRNA injection, mosquitoes were fed on a mouse infected with GFP-expressing parasites. Mosquitoes were dissected 8 to 10 d later to gauge prevalence of infection and mean oocyst numbers per midgut (Figure 1A, Figure S3A). As reported earlier, *Lp* silencing strongly reduced the number of developing oocysts. Strikingly, silencing *TEP1* at the same time as *Lp* annihilated the effect of *Lp* silencing, i.e. yielded the high oocyst numbers typically observed upon silencing of *TEP1* alone. Therefore, the low oocyst counts observed in Lp-depleted mosquitoes are not due to a nutritional dependence of ookinetes on Lp-derived lipids but are a consequence of TEP1 activity. This result also suggests that the increased parasite killing in Lp-depleted mosquitoes takes place at the ookinete stage, since TEP1 binding does not kill oocysts. Further, these results imply that the loss of Lp renders ookinetes more vulnerable to TEP1-dependent killing.

To explain these data, we initially hypothesized that Lp particles might physically sequester components of the TEP1 machinery in an inactive state, but a search for Lp-associated immune factors was unsuccessful (with the notable exception of prophenoloxidase), suggesting that TEP1-containing complexes are not carried in the hemolymph by Lp particles (see Text S1 and Figure S1).

To investigate whether the adverse effect on immunity is a specific property of Lp or may be manifested as well by other nutrient transport factors, we injected mosquitoes with *dsVg* and compared parasite development with *dsLacZ* and *dsTEP1*-injected mosquitoes. A 4-fold reduction in mean parasite numbers was observed in the *dsVg* group compared to *dsLacZ* controls (*p*<0.001, *p*<0.001, *p*<0.05, and *p*<0.05 depending on the replicate of this experiment; Figure 1B and Figure S3B). This effect was more profound than the effect of *dsLp* (Figure 1A and 1E). We then examined whether depletion of the major yolk protein would compromise oogenesis. In contrast to *Lp* silencing, which resulted in total abortion of ovary development, roughly 50% of mosquito females still developed eggs after silencing of *Vg* compared to 80% in *dsLacZ* control mosquitoes (Figure 1C), though ovaries that did mature usually contained only a few eggs bearing melanotic spots (unpublished data). When given a chance to lay, Vg-silenced females did lay a few eggs, the majority of which never hatched (unpublished data). The difference in strength between the *Lp* and *Vg* silencing phenotypes regarding egg development suggests either that Lp is more crucial than Vg for egg development or that the efficiency of *Lp* silencing is greater than the efficiency of *Vg* silencing. Residual Vg protein may allow the development of a few eggs in *dsVg*-treated mosquitoes. It is interesting to note that the strengths of the silencing phenotypes are reversed when considering parasite survival. To verify the efficiency of RNAi-mediated depletion of Lp and Vg, we used specific antibodies directed against the large and small subunits of Lp, and against Vg. RNAi silencing caused Lp and Vg protein amounts to drop below 10% of control levels (Figure S2). Subsequently, we systematically controlled for *Lp* and *Vg* silencing efficiency and noted that Vg depletion was somewhat more variable than Lp depletion, residual Vg protein sometimes approaching 20% of control levels (unpublished data). Strikingly, this analysis revealed that the major protein bands detected in hemolymph samples by Coomassie staining of SDS-PAGE gels (or of PVDF membranes after protein transfer) correspond to the Vg and Lp signals detected by specific antibodies (Figure S2). We excised these easily visualized bands from Coomassie-stained protein gels and submitted them to MALDI mass spectrometry. The peptide mass spectra were searched against the NCBInr database. Each band from a triplet running between 160 and 200 kDa was unequivocally identified as Vg, and the bands running at ∼250 and 80 kDa were unequivocally identified as the large and small subunits of Lp, respectively. In addition, a protein running at ∼70 kD and showing an expression pattern identical to that of the ∼200 kD Vg band (including after RNAi silencing) was identified as the N-terminal fragment of the polypeptide encoded by *Vg* mRNA (visible in Figures 3C, 4C, and S1). This fragment was not recognized by our Vg antibody, raised against a C-terminal Vg fragment. Its existence is consistent with the cleavage of *Ae. aegypti* Vg prior to secretion [33]–[35]. No contaminating proteins were detected at these sizes in the mass spectrometry analysis. Therefore, Lp and Vg proteins can be readily visualized after hemolymph electrophoresis and Coomassie staining of SDS-PAGE gels even without immunoblotting. The efficiency of *TEP1* silencing was also confirmed by immunoblotting (Figure 1D).

**Figure 2:**
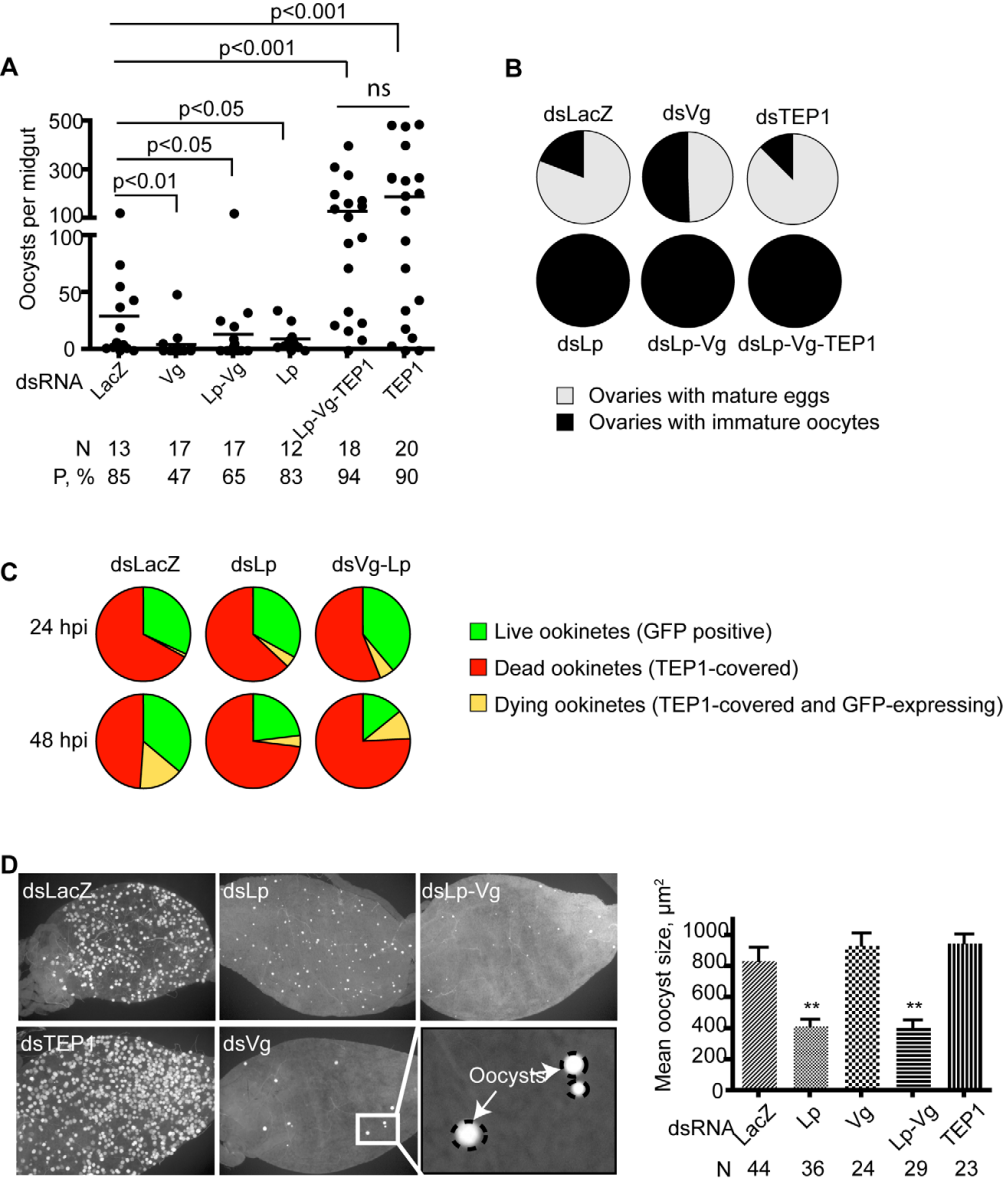
Vg and Lp are involved in TEP1-dependent parasite killing. Mosquitoes were injected with the indicated combinations of *dsLacZ*, *dsVg*, *dsLp*, *dsTEP1*. (A) Parasite counts. *TEP1* silencing rescues the effect of Vg depletion on parasite loss. (B) Oogenesis. *TEP1* knockdown doesn't rescue the effect of Lp/Vg depletion on oogenesis. (C) Mosquitoes infected with *P. berghei* were dissected 24 and 48 h post blood meal. Midguts were fixed and immunostained with anti-TEP1 antibodies. The percentages of live GFP-expressing parasites (green), dying parasites (GFP-positive but partly covered by TEP1), and dead parasites (GFP negative, TEP1-covered) were determined on microscope images. 48 hpi, the percentage of dead, TEP1-labelled ookinetes is markedly higher in *dsLp* or *dsLp*-*Vg* mosquitoes than in the *dsLacZ* controls. In the LacZ control, the percentage of live parasites increases at 48 h because of the progressive clearance of already dead parasites. See Table S1 for parasite numbers scored in each of three independent repeats of this experiment. (D) Lp is required for oocyst maturation. Parasite development was gauged 8 dpi by estimating the size of oocysts in mosquitoes after the depletion of *Lp*, *Vg*, or double KD *Lp-Vg* compared to *TEP1* and *LacZ* knockdown controls. Pictures of dissected midguts were analyzed using Axiovision. Parasite sizes were estimated by the surface area of each individual oocyst and averaged as mean oocyst size per *ds*RNA treatment, yielding the graph to the right. Lp depletion alone or with Vg significantly reduced oocyst sizes compared to controls.

We next investigated whether Vg and Lp cooperate to sustain oogenesis and parasite development or are involved in independent processes. We performed double-knockdown experiments by simultaneously injecting *dsVg-dsLp* to compare to single injections of *dsVg* and *dsLp* as controls. As expected, *dsLp* completely blocked oogenesis and the same was observed in concomitant *dsLp-dsVg* knockdowns (Figure 1F). Moreover, single *dsVg* (*p* = 0.0001) and double *dsLp-dsVg* (*p*<0.0001) knockdowns caused comparable reductions in oocyst counts; these reductions in oocyst numbers were stronger than in the single *dsLp* knockdown (*p* = 0.024) (Figure 1E). These results suggest that the influences of Lp and Vg on reproduction and immunity are balanced differently. Lp may be more crucial for oogenesis than Vg, whereas Vg influences *Plasmodium* survival more strongly than does Lp. In most experiments, the effect of *Vg* and *Lp* knockdowns on parasite counts did not appear to be additive (Figures 1E, 2A, and unpublished data). Although this observation is not supported by strong statistical significance, it raises the possibility that the two proteins may be involved in a single process benefiting ookinete survival in the physiological situation.

To determine whether similarly to Lp, the effect of Vg on parasite development required TEP1 function, we performed triple knockdown experiments by injecting combinations of *dsTEP1*, *dsVg*, *dsLp*, or control *dsLacZ*. Again, total inhibition of oogenesis was observed in all dsRNA combinations that included *dsLp*, suggesting that oogenesis is not influenced by TEP1 function but absolutely requires Lp (Figure 2B). In striking contrast, high parasite loads similar to that detected in the *dsTEP1* single knockdown were obtained when TEP1 was depleted simultaneously to Vg (unpublished data) or to both Vg and Lp (Figure 2A, Figure S3C). These findings imply that blocking the transport of lipids and vitellogenin-derived nutrients does not limit parasite survival when the immune defense is suppressed; instead, the observed reduction in parasite numbers in *dsLp* and *dsVg* knockdowns is dependent on TEP1. We conclude that TEP1-dependent parasite killing is more efficient when Lp and/or Vg levels are low and that the TEP1-mediated immune pressure exerted by the vector is a bigger impediment to the establishment of a *Plasmodium* infection than nutrient availability. If this constraint is removed via TEP1 depletion, *Plasmodium* parasites can effectively exploit even reduced vector resources and proceed with the formation of viable oocysts.

We next examined at which level Vg and Lp genetically interact with TEP1. Binding of mature TEP1 to the parasite surface is one of the first steps leading to parasite killing; either increasing or reducing this event greatly influences the outcome of infection [31],[36]. Therefore, we gauged the efficiency of TEP1 binding to ookinetes in *dsLp-* and *dsLp-Vg*-injected mosquitoes. At early time points (24 hpi) TEP1 binding to ookinetes did not differ in the Lp or Lp-Vg -depleted versus control mosquitoes; but at 48 hpi 70% to 86% of ookinetes were TEP1 positive (i.e., either dead or moribund) in *dsLp-* or *dsVg-Lp*-injected mosquitoes versus only 41% to 68% in *dsLacZ* controls (Figure 2C and Table S1, *p* = 0.005 or less by chi-square analysis). Thus, TEP1 binding to parasites is more efficient in the absence of Lp/Vg. This strongly suggests that physiological levels of Vg and Lp interfere with the efficient binding of TEP1 to ookinetes once the invasion phase is completed.

To see if we could also detect an effect of Lp and Vg depletion at a later stage of parasite development, we examined oocyst growth. Strikingly, oocyst size 9 d after infection was markedly reduced when Lp, but not Vg, was depleted (Figure 2D). In contrast to oocyst numbers, silencing *TEP1* at the same time as *Lp* did not rescue oocyst growth (unpublished data), indicating that the small oocyst size does not result from TEP1 activity in Lp-deficient mosquitoes. This supports the hypothesis that Lp contributes nutrients to oocyst development [26]. Therefore, Lp benefits *Plasmodium* development at two independent levels: an early effect favoring ookinete survival by protecting against TEP1-dependent killing, and a later effect favoring normal oocyst growth. The latter effect does not require Vg or TEP1 function.

### Vg and Lp Do Not Affect *TEP1* Expression or Cleavage, but Lp Is Necessary for Proper *Vg* Expression

**Figure 3:**
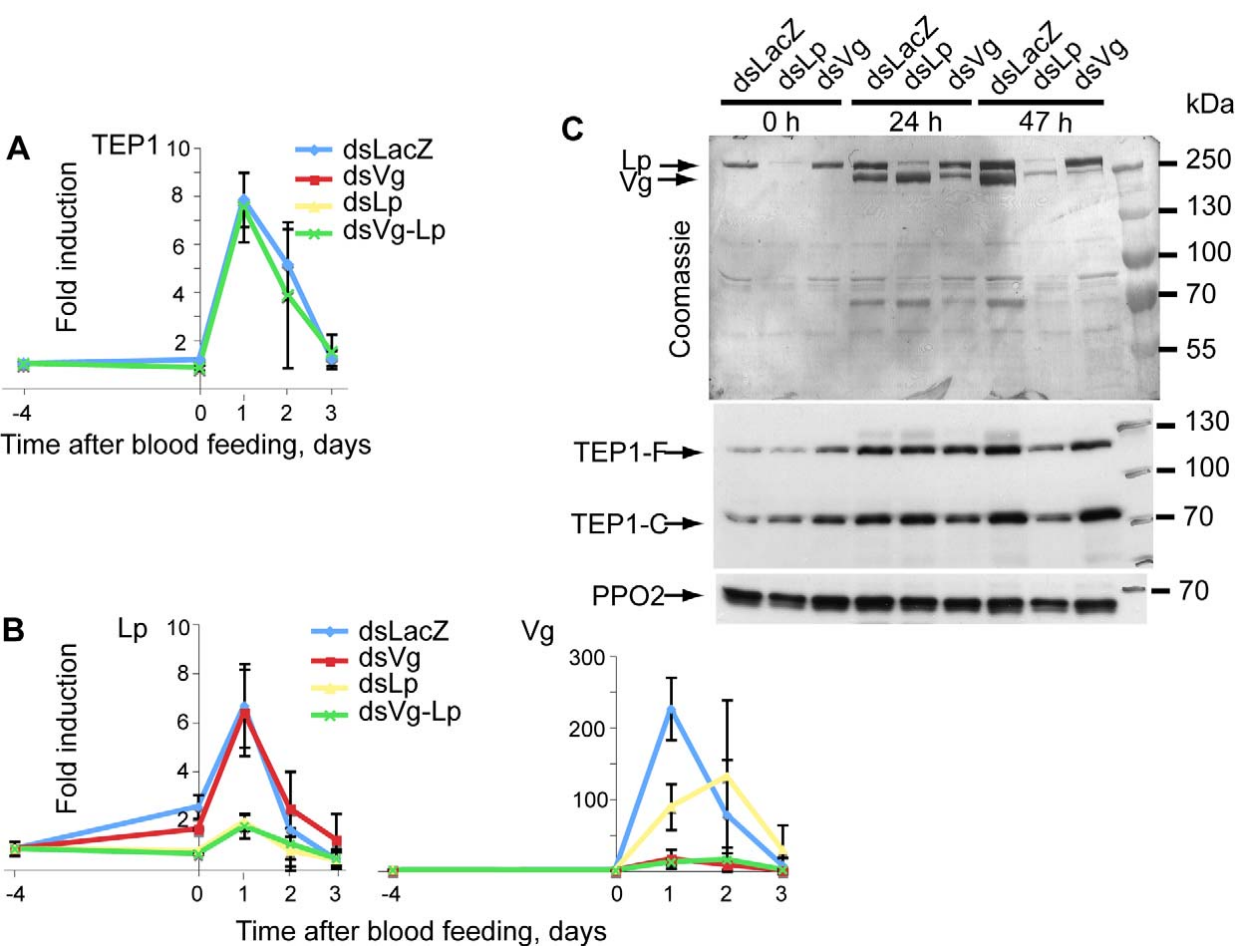
Lp is required for normal Vg expression. Mosquitoes were injected with *dsLp*, *dsVg*, or *dsLp*+*dsVg*. (A, B) *TEP1*, *Vg*, and *Lp* expression, respectively, was measured at several time points after *P. berghei* infection using quantitative RT-PCR. (C) Lp and Vg protein levels in mosquito hemolymph were gauged by Coomassie staining; TEP1 (full length and processed) by immunoblotting. PPO2 served as a loading control. Note that levels of Vg protein are strongly reduced at 47, but not 24, h after infection specifically in *dsLp*-treated mosquitoes.

Previous work [7],[31] has demonstrated that boosting mosquito basal immunity via depletion of the inhibitory IκB protein Cactus up-regulates components of the TEP1 pathway (including TEP1, LRIM1, and APL1C) and completely blocks parasite development. Therefore, we asked whether the knockdown of *Vg* and *Lp* could mimic the effect of Cactus depletion and elevate *TEP1* expression levels, providing an explanation to the above observations. We silenced *Lp* and/or *Vg* and examined the transcript levels of *TEP1* before and after blood feeding using quantitative real-time polymerase chain reaction (qRT-PCR). Silencing of the two nutrient transport genes did not alter *TEP1* expression (Figure 3A). We then evaluated the effect of *Lp* and *Vg* silencing on TEP1 protein amounts and TEP1 cleavage in the hemolymph by immunoblotting using polyclonal anti-TEP1 antibodies. This analysis did not reveal any marked increase in the amounts of full-length or mature TEP1 protein (Figures 3C and 1D).

Surprisingly, silencing of *Lp* reproducibly lowered the expression of *Vg* mRNA (Figure 3B and unpublished data). At the protein level, Lp depletion strongly reduced Vg levels at 47 h (but not 24 h) post-infectious feeding compared with the controls (Figure 3C), confirming that Lp is indeed required for full *Vg* expression between day 1 and day 2 post-infectious blood-feeding. In contrast, the depletion of Vg had no effect on *Lp* expression (Figure 3B) or protein levels (Figure 3C).

### Depletion of *Cactus* Represses *Vg* Expression

**Figure 4:**
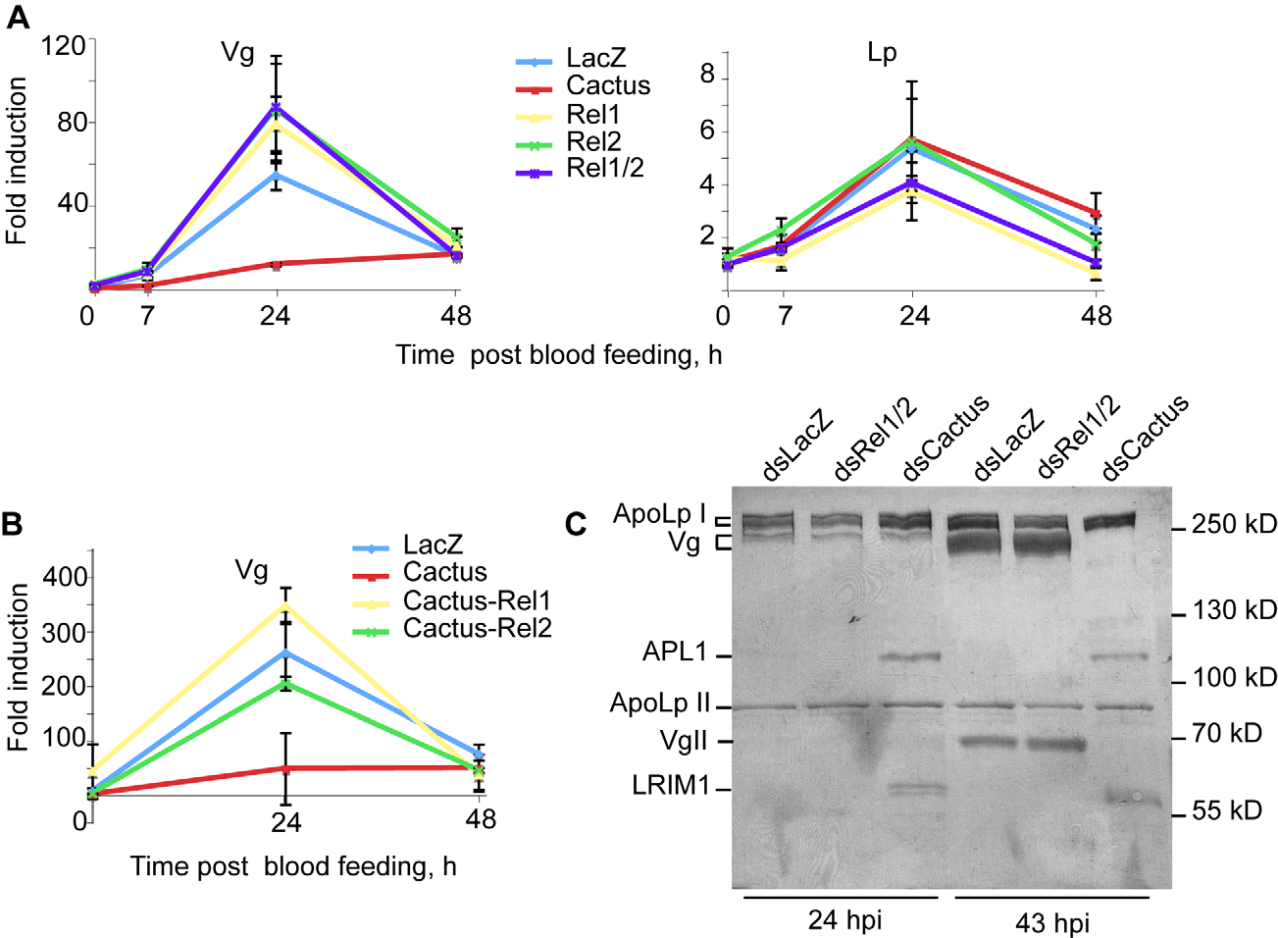
*Vg* expression is repressed by NF-κB factors REL1/REL2. Mosquitoes were injected with the indicated combinations of *dsCactus*, *dsRel1*, *dsRel2*, and the expression levels of *Vg* and *Lp* examined by qRT-PCR at the indicated time points after infection and compared to *dsLacZ* control. Gene expression is expressed relative to the LacZ control at time 0. (A) *Vg* expression was inhibited in *dsCactus* but increased in *dsRel1/Rel2* mosquitoes. (B) Concomitant depletion of Cactus and Rel1 restored *Vg* expression. (C) Coomassie staining of hemolymph proteins after electrophoresis on a 7% SDS-PAGE gel and transfer to a PVDF membrane. No change in Vg protein is seen 24 h after infection, but at 43 h *dsCactus* completely blocks Vg expression. Protein identities are indicated to the left. The identity of the subunits of Lp and Vg, and the identity of APL1C and LRIM1 proteins were established by mass spectrometry of the Coomassie-stained bands and by immunoblotting. APL1C and LRIM1 over-expression confirms the efficiency of *Cactus* silencing.

The unexpected observation that *Lp* and *Vg* knockdown simultaneously arrests oogenesis and facilitates TEP1 binding to ookinetes led us to re-examine the previously observed striking phenotype of *dsCactus*, which boosts basal immunity while arresting oogenesis ([31] and unpublished data). Depleting the IκB-like repressor protein Cactus increases the activity of NF-κB factors REL1 and REL2, leading to elevated expression of *TEP1* and other immune factors. Therefore, we investigated whether REL1, REL2, and Cactus influence the expression of *Vg* and/or *Lp*. To this end, mosquitoes were injected with either *dsRel1*, *dsRel2*, *dsCactus*, or co-injected with *dsRel1-dsRel2*, *dsRel1-dsCactus*, *dsRel2-dsCactus*, and *dsLacZ* control. Mosquitoes were fed on an infected mouse, and subsequently, the expression of *Vg* and *Lp* was monitored by qRT-PCR. Strikingly, *Vg* expression was almost abolished in *dsCactus* mosquitoes at 24 hpi; conversely, the depletion of REL1 or REL2 at this time point elevated *Vg* expression above the levels in the *dsLacZ* control (Figure 4A). Interestingly, concomitant silencing of *Cactus/Rel1* and *Cactus/Rel2* restored *Vg* expression to physiological levels (Figure 4B), indicating that REL1 and REL2 contribute to the regulation of *Vg* expression. At the protein level, Vg amounts were unchanged at 24 h but strongly reduced 43 h after infectious blood feeding specifically in *dsCactus*-injected-mosquitoes (Figure 4C), confirming the qPCR data and revealing a clear delay between mRNA and protein fluctuations. Thus, in the *dsCactus* background, while *TEP1* expression is upregulated, *Vg* expression is directly or indirectly repressed by REL1/2. Therefore, the Cactus protein affects TEP1 and Vg levels in opposite directions. We extended our analysis to Lp, but in contrast to the situation reported for *Ae. aegypti* [24], its expression was unaffected by the knockdown of the NF-κB-like factors (Figure 4A). Since *Vg* silencing alone, unlike *Cactus* silencing, is not sufficient to completely block oogenesis, other molecules required by developing mosquito oocytes may be regulated by Cactus in the same manner as Vg.

Taken together, our findings uncover the complex phenotype of Cactus depletion. It leads to a lower level of *Vg* expression after a blood meal, thereby contributing to the arrest in oogenesis seen in *Cactus* knockdown mosquitoes. On the other hand, it stimulates the mosquito antiparasitic defense at least at two different levels: (i) by lowering the level of Vg, rendering TEP1-mediated killing more efficient, and (ii) by elevating the levels of TEP1 pathway proteins.

## Discussion

The first indication that nutrient transport after a blood meal influences mosquito susceptibility to *P. berghei* was provided by Vlachou et al. [23], who demonstrated that experimental depletion of the lipid carrier protein Lp by RNAi reduces the number of developing oocysts in the mosquito midgut. Recently, these results were extended to *P. falciparum* [25]. However, how and at which stage of development the parasites were eliminated in Lp-deficient mosquitoes remained to be determined. We show here that the major yolk protein Vg shows a similar but more drastic knockdown phenotype than Lp on *Plasmodium* survival and that the Lp and Vg depletion phenotypes require the function of the immune factor TEP1, which targets ookinetes for killing. Further, high numbers of parasites actually survive and turn into oocysts even in the context of Lp and/or Vg depletion, as long as TEP1 is also experimentally depleted. From these observations, we infer that physiological levels of both nutrient transport proteins following a blood meal somehow dampen the strength of the immune defense and protect ookinetes against destruction by the TEP1 pathway. The effects of Lp and Vg depletion on TEP1-mediated parasite killing are similar, and we find that Lp is required for the full induction of *Vg* expression on day 2 following an infectious blood meal. We therefore propose that Lp may indirectly affect ookinete survival by influencing *Vg* expression, while Vg impinges either directly or more closely than Lp on the TEP1-killing mechanism.

The induction of *Vg* expression after a blood meal requires both the TOR pathway and ecdysone signaling [14]. It is unclear why Lp depletion reduces the expression of *Vg* after an infectious blood meal. One possible explanation is that an Lp shortage precludes ovarian follicle development, preventing the normal secretion of ecdysone by follicle cells; thus leading to the reduction in Vg expression. However, attempts to rescue the *Lp* silencing effect on *Vg* expression with exogenously provided 20-hydroxyecdysone were unsuccessful. As the lower level of *Vg* expression in Lp-deficient *A. gambiae* is reminiscent of the situation observed in adult *Ae. aegypti* mosquitoes malnourished during larval life [37], it would be interesting to determine if *Plasmodium* survival is compromised in such malnourished mosquitoes in laboratory and field settings.

The GFP-tagged *P. berghei* strain used in this study provides a good model and enables analyses of vectorial capacity that are much more demanding with wild malaria parasites. However, recent studies indicate that the mosquito response to *P. berghei* and to *P. falciparum* differ in important ways [10],[38]. In addition, the *P. berghei–A. gambiae* model is an unnatural host-parasite association. Therefore, it will be important to see whether our observations hold true in the *A. gambiae–P. falciparum* relationship. Importantly though, the TEP1 pathway does limit *P. falciparum* survival in *A. gambiae* natural infections ([39] and Levashina et al., unpublished results) and the Lp knockdown was shown to have similar effects in both systems [25].

What is the molecular basis of the negative effect of the two nutrient transport proteins on the TEP1 pathway? We initially hypothesized that Lp-scaffolded lipidic particles could sequester components of the TEP1 pathway in an inactive state. However, TEP1 and its interacting partners LRIM1 and APL1C were not detectable in Lp extracts, suggesting that the *Plasmodium*-killing machinery is not carried by Lp particles. Instead, RNAi-mediated depletion of Lp and, more strikingly, of Vg resulted in more efficient TEP1 binding to the surface of ookinetes at 48 hpi, promoting their killing. One explanation could be that Vg (and perhaps Lp, to a lesser extent) are recruited to the parasite surface, where they might mask TEP1 binding sites. Consistent with this idea, fish vitellogenin has recently been found to bind microorganisms and to opsonize them for phagocytosis [40]. Mosquito Vg may behave non-productively in a similar manner and outcompete TEP1 from the ookinete surface. Alternatively, a physical interaction between TEP1 and Vg could inhibit TEP1 activity, a hypothesis that should be further investigated. Yet another possible explanation is that transient interactions of ookinetes with Vg might alter the lipid composition in the ookinetes' membrane, rendering them less visible to the TEP1 machinery. The parasite molecules to which TEP1 covalently attaches are currently unknown, but hydroxyl residues on surface lipids could be good targets for thioester-dependent TEP1 covalent binding.

We further observed a retarded oocyst growth in *Lp*-deficient mosquitoes 9 d post infection. This phenotype was specific to Lp, as parasites developed normally in *Vg*-deficient mosquitoes. Therefore, Lp is a probable lipid source for developing oocysts. Indeed, Lp was detected inside *P. gallinaceum* oocysts in vitro, suggesting that oocysts tap some of the host's Lp for their development [26]. Taken together, Lp appears to regulate parasite development at two distinct stages by two independent mechanisms: (i) providing an indirect protection to ookinetes via regulation of Vg levels after a blood meal and thereby dampening TEP1 binding to ookinetes, and (ii) exerting a direct nutritional role by supplying lipids to growing oocysts.

The quantitative RT-PCR and protein expression results reported here added the IκB/NF-κB-like factors Cactus/REL1 and REL2, previously known to control immunity [29]–[31], to the list of factors that influence *Vg* expression. We propose that Cactus depletion boosts TEP1 parasite killing by simultaneously increasing *TEP1* expression [31] and decreasing the expression of *Vg*, in the absence of which TEP1-mediated killing is more efficient. Previously, the reason why Cactus depletion blocked oogenesis while boosting anti-*Plasmodium* immunity was unknown. Our results shed new light on this phenomenon by suggesting that Cactus activity is necessary for the expression of *Vg*, and probably of additional factors involved in vitellogenesis.

Although many mosquito genes showing antiparasitic activity are induced by the NF-κB-like factors REL1 and REL2 [12],[29]–[31],[41], it is currently unclear whether parasite invasion of mosquito tissues actually activates the NF-κB pathways. However, the expression of nutrient transport molecules is affected by signals arising from the parasite's invasion, in addition to being influenced by hormone signaling, the TOR pathway, and NF-κB factors. Indeed, ookinete invasion of the midgut induces *Lp* mRNA expression further than does an uninfected blood meal in *A. gambiae* and *Ae. aegypti* [23],[24]. At the protein level, we did not observe a corresponding increase in Lp amounts using specific antibodies (unpublished data), which may reflect consumption of the additionally produced Lp by parasites and/or by the midgut wound healing response to parasite invasion. This implies that Lp protein homeostasis is under tight physiological regulation. Conversely, Ahmed et al. [42] reported that parasite invasion reduces the abundance of the *Vg* transcript in *A. gambiae*, while Vg protein levels were only transiently reduced before accumulating in the hemolymph. Therefore, the production of both proteins is subjected to multiple physiological switches. The reported changes in Vg levels correlated with apoptosis of patches of ovarian follicular cells, which was prominent following infections and immune stimulation. Dying ovarian follicles stop secreting ecdysteroids and taking up Vg protein, which may explain both the drop in *Vg* transcription and the accumulation of Vg protein in the hemolymph [43],[44]. It would be interesting to identify infection-dependent signals arising at the midgut and triggering ovarian follicle apoptosis. In *Drosophila*, pathogenesis is also reported to trigger cell death in ovaries [45]. In the presence or absence of an infection, activation of the Immune deficiency (Imd) pathway (e.g., by injection of dead bacteria) negatively impacted oogenesis. This effect depended on the immune status, as oogenesis remained normal in Imd pathway mutants injected with dead bacteria [46]. The mosquito Cactus/REL1/REL2 NF-κB pathway is related to the *Drosophila* Toll and Imd immune pathways; its targets would therefore represent attractive candidates as modulators of mosquito reproduction. A full understanding of the interactions between reproductive and immune functions in mosquitoes will require a thorough study of the molecular pathways influencing the transcription of immune and vitellogenic factors, and how these pathways are affected by blood meals, immune defense, and parasite invasion. To our knowledge, Vg and Cactus are the first molecules reported to occupy a central position at the interface between reproduction and immunity, providing a molecular handle to further explore the long-suspected trade-off between these two processes.

## Material and Methods

### Potassium Bromide Gradient Purification of Lipophorin Particles

Approximately 0.5 g of mosquito adults (ca. 330 mosquitoes) were roughly ground with a Polytron electric homogenizer in 2 ml ice-cold TNE buffer (100 mM Tris-HCl pH 7.5, 0.2 mM EGTA, 150 mM NaCl) + Complete protease inhibitors (Roche). Debris were centrifuged at 4°C in a tabletop centrifuge. The supernatant was transferred to 2.2 ml ultracentrifuge tubes and spun for 3 h at 120,000 g at 4°C in a Sorvall ultracentrifuge equipped with an S55-S rotor. The cleared supernatant was recovered, completed with solid potassium bromide to a final concentration of 0.34 g/ml, overlayed with 0.5 ml TNE buffer+0.33 g/ml KBr, and centrifuged in 2.2 ml PET ultracentrifuge tubes (Hitachi Koki) at 250,000 g, 10°C, for at least 36 h. The top layer of fat was discarded and 5 or 6 fractions of 0.5 ml were carefully collected starting from the top. Lipophorin particles were present in the top fraction, while the majority of other proteins fractionated into the fourth.

### Lipophorin and Vitellogenin Antibodies

The top fraction of a potassium bromide gradient prepared using a scale-up of the above method was desalted on a Pharmacia PD-10 column according to the manufacturer's instructions. The two subunits of Lp were the predominant proteins in the extract according to Coomassie staining of an SDS-PAGE gel. Protein amount was quantified with a Bradford assay. Six-week-old female BALB/c mice were injected intraperitoneally with 40 µg of these lipophorin particles and 100 µg of poly I/C as adjuvant. Three injections were performed at 2-wk intervals. Four days prior to hybridoma fusion, mice with positively reacting sera were reinjected. Spleen cells were fused with Sp2/0.Agl4 myeloma cells as described [47]. Hybridoma culture supernatants were tested at day 10 by ELISA for cross-reaction with purified Lp particles. Positive supernatants were then tested by Western blot on mosquito extracts. All ELISA-positive supernatants recognized peptides corresponding in size to either the large (250 kDa) or the small (80 kDa) Lp subunit. Specific cultures were cloned twice on soft agar. A hybridoma clone (2H5, immunoglobulin subclass IgG2aκ) recognizing the 80 kDa Lp subunit was selected and ascites fluid was prepared by injection of 2×10^6^ hybridoma cells into pristane-primed BALB/c mice. The resulting antibody efficiently immuno-precipitated the 80 kDa Lp subunit and co-immunoprecipated the 250 kDa subunit. The identity of both immuno-precipitated subunits, excised from Coomassie-stained protein gels, was confirmed by mass spectrometry. Similarly, we prepared a monoclonal antibody (2C6) recognizing the large Lp subunit. Rabbit polyclonal antibodies specific to Vg were obtained by immunizing rabbits with a purified recombinant Vg fragment fused to GST. The *Vg* gene fragment used for protein production was amplified from mosquito cDNA using attB-site (capital letters)-containing primers GGGGACAAGTTTGTACAAAAAAGCAGGCTtcaagtttgtgctgcagcacaagcag and GGGGACCACTTTGTACAAGAAAGCTGGGTCCTAagcgcaagatggatggtagtttc. The PCR product was cloned into pDEST15 (Invitrogen) using the Gateway technology. Protein was produced in *E. coli* BL21-AI.

### Immunoprecipitation

120 adult mosquitoes were severed by opening the thorax and abdomen cuticles with fine forceps and bled on ice in 1 ml IP buffer (TRIS pH 7.9 50mM, NaCl 100 mM, EDTA 2 mM, BSA 0.1 µg/ml) + Complete protease inhibitors (Roche). Carcasses and cellular debris were removed by two successive 2,500 g centrifugation steps (for 2 min at 4°C); the extract was further cleared by three 16,500 g centrifugations (2 min each). The sample was pre-cleared for 1 h at 4°C under gentle rocking with 2 µg of an irrelevant mouse IgG2aκ antibody that was removed by incubation at 4°C with 35 µl protein A-sepharose slurry (Pharmacia) for 1 h followed by centrifugation. Supernatant was split in two aliquots, one subjected to a 1 h incubation with specific antibody and the other with a non-specific antibody of the same immunoglobulin class. 35 µl of protein A-Sepharose were added to each sample, further rocked at 4°C for 1 h, centrifuged. The supernatant was saved (post-IP supernatant sample). Sepharose beads were washed 5×10 min in TE buffer with or without 500 mM KCl, successively. Lipophorin and associated proteins were eluted from the beads using SDS-PAGE sample buffer and submitted to Western blotting.

### Hemolymph Protein Samples for SDS-PAGE

At least 8 anesthetized mosquitoes were aligned on ice under the binocular microscope. Their proboscis was clipped with dissection scissors. Each mosquito was gently pressed on the thorax with forceps and the hemolymph droplet forming at the tip of the cut proboscis was collected into 1× sample (Laemmli) buffer. An hemolymph amount equivalent to that collected from 4 mosquitoes was loaded in each lane of SDS-PAGE gels.

### RNAi and Infections

The 741 bp long HincII fragment of *Vg1* (AGAP004203) and the 431 bp long *Bsp*HI/*Bsg*I fragment of *Lp* (AGAP001826) were cloned from cDNA library clones into the pLL10 vector. RNAi constructs for TEP1 and NF-κB factors have been described (Frolet et al. 2006) [31]. Potential cross-silencing effects of the chosen sequences were analyzed using the Deqor software ([48]; http://deqor.mpi-cbg.de/) with the predicted *A. gambiae* transcriptome ENSEMBL database. DsRNA was synthesized as previously described [36]. *A. gambiae* susceptible G3 strain were maintained at 28°C, 75%–80% humidity, and a 12/12 h light/dark cycle. Two-day-emerged adult female mosquitoes from the same cohort were injected with 0.2 µg of dsRNA using a Nanoject II injector (Drummond, http://www.drummondsci.com). Co-injection experiments were performed by injecting a double volume of 1∶1 mixtures of 3 µg/µl solutions of dsRNAs. Four days after dsRNA injection mosquitoes were fed on a mouse carrying *P. berghei* GFP-con 259cl2 as previously described [36],[37]. Statistical significance was determined with a Kruskall-Wallis test for non-parametric data followed by Dunn's post-test. The indicated *p* values are those obtained with Dunn's test.

### Assessment of Ovary Development

The ovaries of dissected females were observed under the binocular microscope. Ovaries containing 3 fully grown eggs or more were scored as positive. Ovaries with only undeveloped oocytes or less than 3 fully grown eggs were scored negative.

### *qRT-PCR*

Total RNA from 10 mosquitoes was extracted with Trizol reagent (Invitrogen) before and after dsRNA injection or after blood feeding. 2–8 µg of RNA was reverse transcribed using M-MLV enzyme and random primers (Invitrogen). Specific primers (Table 1) were used at 300 nM for qRT-PCR reactions. Ribosomal protein L19 (RPL19) served as an internal control to normalize gene expression. The reactions were run on an Applied Biosystems 7500 Fast Real-Time PCR System using Power SYBR Green Mastermix (http://www.appliedbiosystems.com).

**Table 1:** Primers used for qRT-PCR.

Gene	Primers for qRT-PCR
*TEP1*	AAAGCTACGAATTTGTTGCGTCA TTCTCCCACACACCAAACGAA
*Vg*	CCGACTACGACCAGGACTTC CTTCCGGCGTAGTAGACGAA
*Lp*	CAGCCAGGATGGTGAGCTTAA CACCAGCACCTTGGCGTT
*RPL19*	CCAACTCGCGACAAAACATTC ACCGGCTTCTTGATGATCAGA

### Fluorescence Microscopy

In order to count the surviving GFP-expressing parasites, mosquito midguts were dissected between 7 and 10 dpi and prepared as previously described [36],[37] and observed under a fluorescence microscope. To assess TEP1 binding to ookinetes, mosquito midguts were dissected at 18, 24, and 48 hpi, fixed in 4% formaldehyde at room temperature for 45 min, then washed with phosphate buffered saline, and stained with anti-TEP1 antibodies as previously described [31],[36]. Parasite numbers and TEP1 labeling were scored using a Zeiss fluorescence microscope (Axiovert 200M) equipped with a Zeiss Apotome module (http://www.zeiss.com). GFP-expressing parasites were considered live while dead parasites were GFP negative. Differential TEP1 staining on ookinete were gauged at 18, 24, and 48 hpi. At least three independent experiments were conducted per treatment group with a minimum of five mosquito midguts per treatment. For each midgut, all ookinetes visible in 4 fields covering most of the midgut were scored. Table S1 summarizes the ookinete counts from three independent experiments.

### MALDI Mass Spectrometry

Coomassie-stained protein bands excised from SDS-PAGE gels were digested with trypsin. Tryptic peptides eluted from the gel slices were subjected to MALDI mass measurement on an Autoflex III Smartbeam (Bruker-Daltonik GmbH, Bremen, Germany) matrix-assisted laser desorption/ionization time-of-flight mass spectrometer (MALDI-TOF TOF) used in reflector positive mode. The resulting peptide mass fingerprinting data and peptide fragment fingerprinting data were combined by Biotools 3 software (Bruker Daltonik) and transferred to the search engine MASCOT (Matrix Science, London, UK). Peptide mass error was limited to 50 ppm. Proteins were identified by searching data against NCBI non-redundant protein sequence database.

## Supporting Information

Figure S1 **Prophenoloxidase but not TEP1 or LRIM1 associates with Lp particles.** (A, top panel) Coomassie-stained polyacrylamide gel resolving mosquito proteins fractionated on a potassium bromide gradient. Molecular weight standards are indicated on the left. Lp subunits (ApoI and ApoII, circled red in lane 1) are the main proteins detectable in top gradient fractions. Fractions 1, 2, 3 are 10-fold concentrated compared to fractions 4, 5, 6. (A, middle and bottom panel) Western blotting with anti-TEP1 and LRIM1 antibodies reveal TEP1 and LRIM1 proteins only in higher density fractions. TEP1-F, full-length TEP1; TEP1-C, C-terminal TEP1 fragment. (B) Western blotting analysis of KBr fractions using anti-PPO2 antibody. A fraction of PPO fractionates with Lp particles. (C) Immunoblotting analysis of Lp particles purified by immunoprecipitation 0, 4, or 14 d after a *P. berghei* infection (dpi) with mouse anti-Lp (ApoLpII) monoclonal antibody. Non-specific mouse antibody (NS) is used as an immunoprecipitation control. TEP1 does not associate with purified Lp and is found only in post-IP (unbound) supernatants.
(0.92 MB TIF)

Figure S2 **Lp and Vg proteins are readily visualized by Coomassie staining.** Mosquitoes were injected with *dsLacZ*, *dsLp*, or *dsVg* as indicated and offered a blood meal 4 d later to induce Vg expression. Hemolymph was collected 24 h after a blood meal from clipped mosquito proboscises. Hemolymph from the equivalent of 4 mosquitoes as well as 5- and 10-fold dilutions of the control *dsLacZ* hemolymph (2 lanes at the right of the gel) was resolved by electrophoresis on a 7% SDS-PAGE gel and transferred to a PVDF membrane. The membrane was subjected to staining with Coomassie brilliant blue (top panel). Proteins were subsequently revealed with the indicated antibodies (lower panels). Molecular weight markers are indicated on the right. Protein bands revealed by the antibodies superpose perfectly with the protein bands revealed by Coomassie staining. The protein identities were confirmed by a mass spectrometric analysis. The intensities of antibody signals in the 5- and 10-fold diluted sample indicate that residual Vg and Lp protein levels are less than 10% of the control level in the corresponding RNAi samples.
(2.34 MB TIF)

Figure S3 (A) Three additional repeats of the experiment shown in Figure 1A. (See Figure 1A for legend.) (B) Three additional repeats of the experiment shown in Figure 1B. (See Figure 1B for legend.) (C) Two additional repeats of the experiment shown in Figure 2A. (See Figure 2A for legend.)
(0.35 MB TIF)

Table S1 **The table summarizes the parasite scores for three independent repeats of the experiment shown in** **Figure 2C** **.** Shown are parasite percentages in each of the three possible classes (live, GFP positive; dying, GFP + TEP1 positive; dead, TEP1 positive). The total number of ookinetes scored for each treatment group is given in parentheses next to the injected dsRNA. *p* values were obtained by chi-square analysis comparing parasite scores in dsLp and dsLacZ-injected mosquitoes or comparing parasite scores in dsLp-Vg and dsLacZ-injected mosquitoes. For this analysis, we summed all TEP1-positive ookinetes (dead + dying). Figure 2C was generated with Experiment 3.
(0.04 MB XLS)

Text S1 **The supplemental text describes lipophorin particle purification from adult mosquitoes by potassium bromide gradient fractionation or immuno-precipitation and a search for immune factors that co-purify with lipophorin.**
(0.10 MB DOC)

## Abbreviations

dsRNA: double-stranded RNA
ELISA: enzyme-linked immunosorbent assay
GFP: green fluorescent protein
GST: glutathione S-transferase
HDL: high-density lipoprotein
hpi: hours post infection
Imd: Immune deficiency
IP: immuno-precipitation
KBr: potassium bromide
kDa: kiloDalton
Lp: Lipophorin (AGAP001826)
LDL: low-density lipoprotein
LRR: leucine-rich repeat
MALDI: matrix-assisted laser desorption/ionization
PCR: polymerase chain reaction
qRT: quantitative real-time
RNAi: RNA interference
SDS-PAGE: sodium dodecyl sulfate polyacrylamide gel
TEP1: thioester-containing protein 1
TOF: time-of-flight
Vg: Vitellogenin (AGAP004203)
TOR: Target of Rapamycin
