# Supplementary figures and images for: The Major Yolk Protein Vitellogenin Interferes with the Anti-Plasmodium Response in the Malaria Mosquito Anopheles gambiae
Source: PLoS Biol. 2010 Jul 20;8(7):e1000434. doi: 10.1371/journal.pbio.1000434 (PMC2907290; doi:10.1371/journal.pbio.1000434)

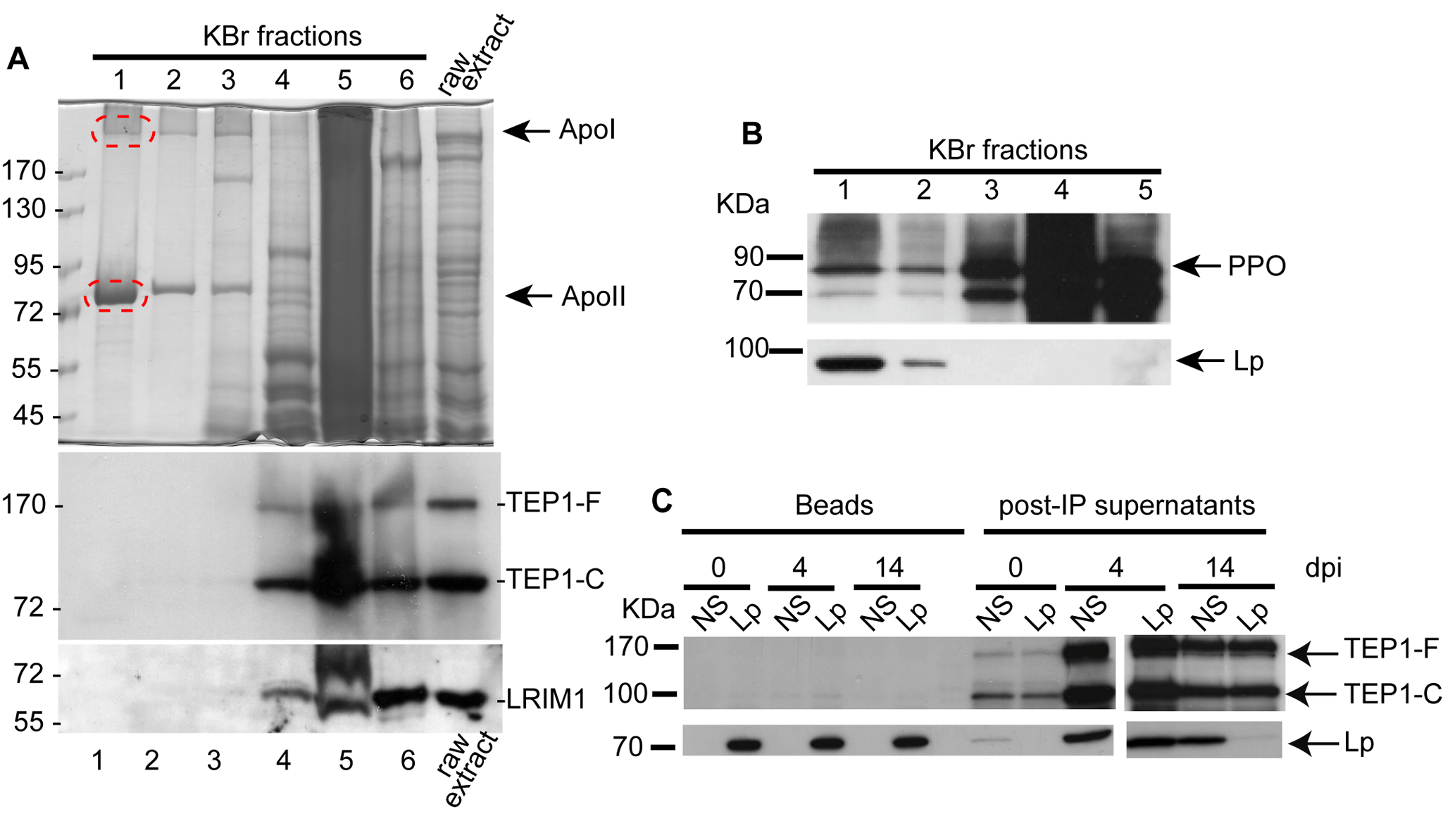

Supplement: Figure S1 — Prophenoloxidase but not TEP1 or LRIM1 associates with Lp particles. (A, top panel) Coomassie-stained polyacrylamide gel resolving mosquito proteins fractionated on a potassium bromide gradient. Molecular weight standards are indicated on the left. Lp subunits (ApoI and ApoII, circled red in lane 1) are the main proteins detectable in top gradient fractions. Fractions 1, 2, 3 are 10-fold concentrated compared to fractions 4, 5, 6. (A, middle and bottom panel) Western blotting with anti-TEP1 and LRIM1 antibodies reveal TEP1 and LRIM1 proteins only in higher density fractions. TEP1-F, full-length TEP1; TEP1-C, C-terminal TEP1 fragment. (B) Western blotting analysis of KBr fractions using anti-PPO2 antibody. A fraction of PPO fractionates with Lp particles. (C) Immunoblotting analysis of Lp particles purified by immunoprecipitation 0, 4, or 14 d after a P. berghei infection (dpi) with mouse anti-Lp (ApoLpII) monoclonal antibody. Non-specific mouse antibody (NS) is used as an immunoprecipitation control. TEP1 does not associate with purified Lp and is found only in post-IP (unbound) supernatants. (0.92 MB TIF) [file pbio.1000434.s001.tif]

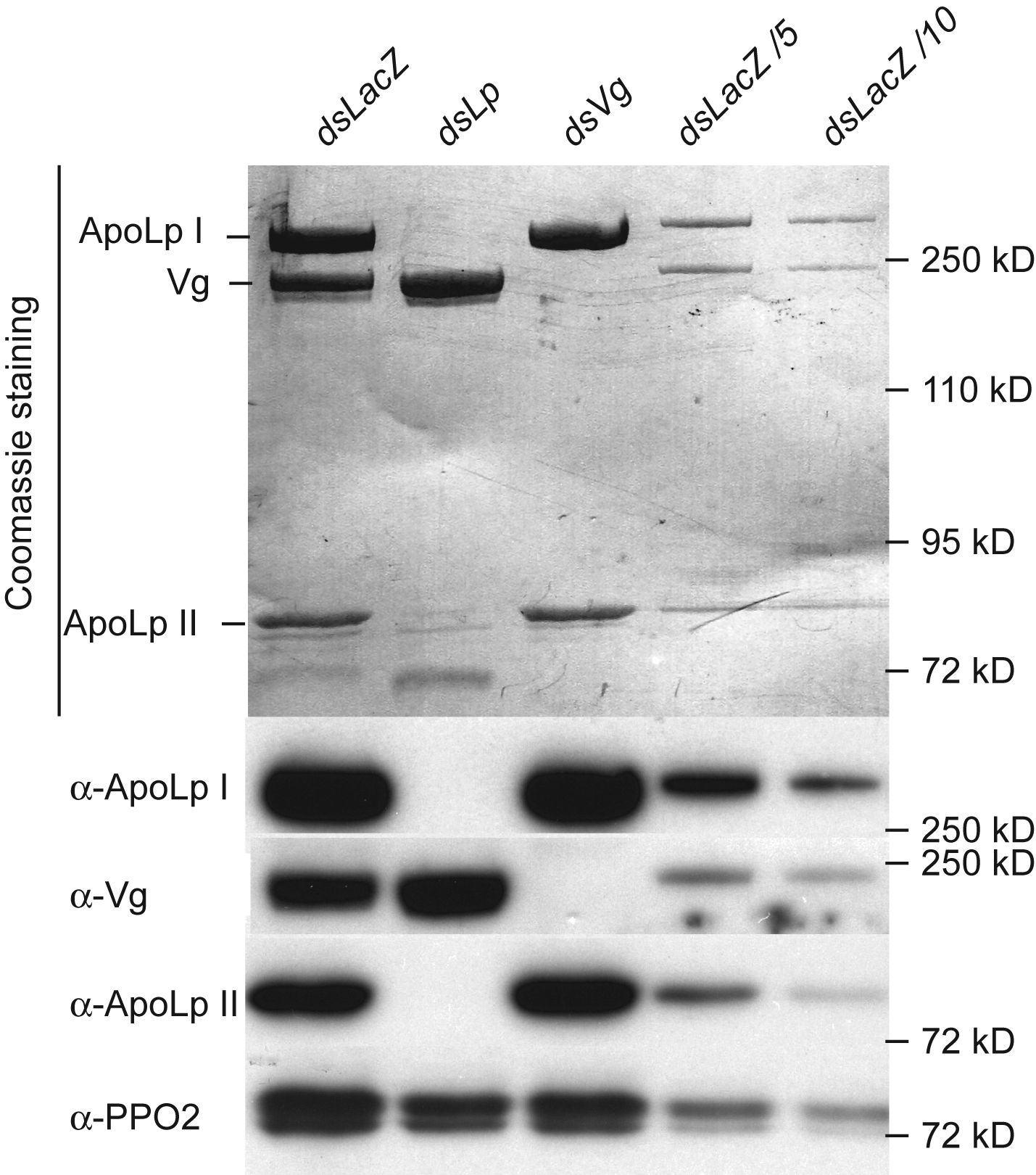

Supplement: Figure S2 — Lp and Vg proteins are readily visualized by Coomassie staining. Mosquitoes were injected with dsLacZ, dsLp, or dsVg as indicated and offered a blood meal 4 d later to induce Vg expression. Hemolymph was collected 24 h after a blood meal from clipped mosquito proboscises. Hemolymph from the equivalent of 4 mosquitoes as well as 5- and 10-fold dilutions of the control dsLacZ hemolymph (2 lanes at the right of the gel) was resolved by electrophoresis on a 7% SDS-PAGE gel and transferred to a PVDF membrane. The membrane was subjected to staining with Coomassie brilliant blue (top panel). Proteins were subsequently revealed with the indicated antibodies (lower panels). Molecular weight markers are indicated on the right. Protein bands revealed by the antibodies superpose perfectly with the protein bands revealed by Coomassie staining. The protein identities were confirmed by a mass spectrometric analysis. The intensities of antibody signals in the 5- and 10-fold diluted sample indicate that residual Vg and Lp protein levels are less than 10% of the control level in the corresponding RNAi samples. (2.34 MB TIF) [file pbio.1000434.s002.tif]

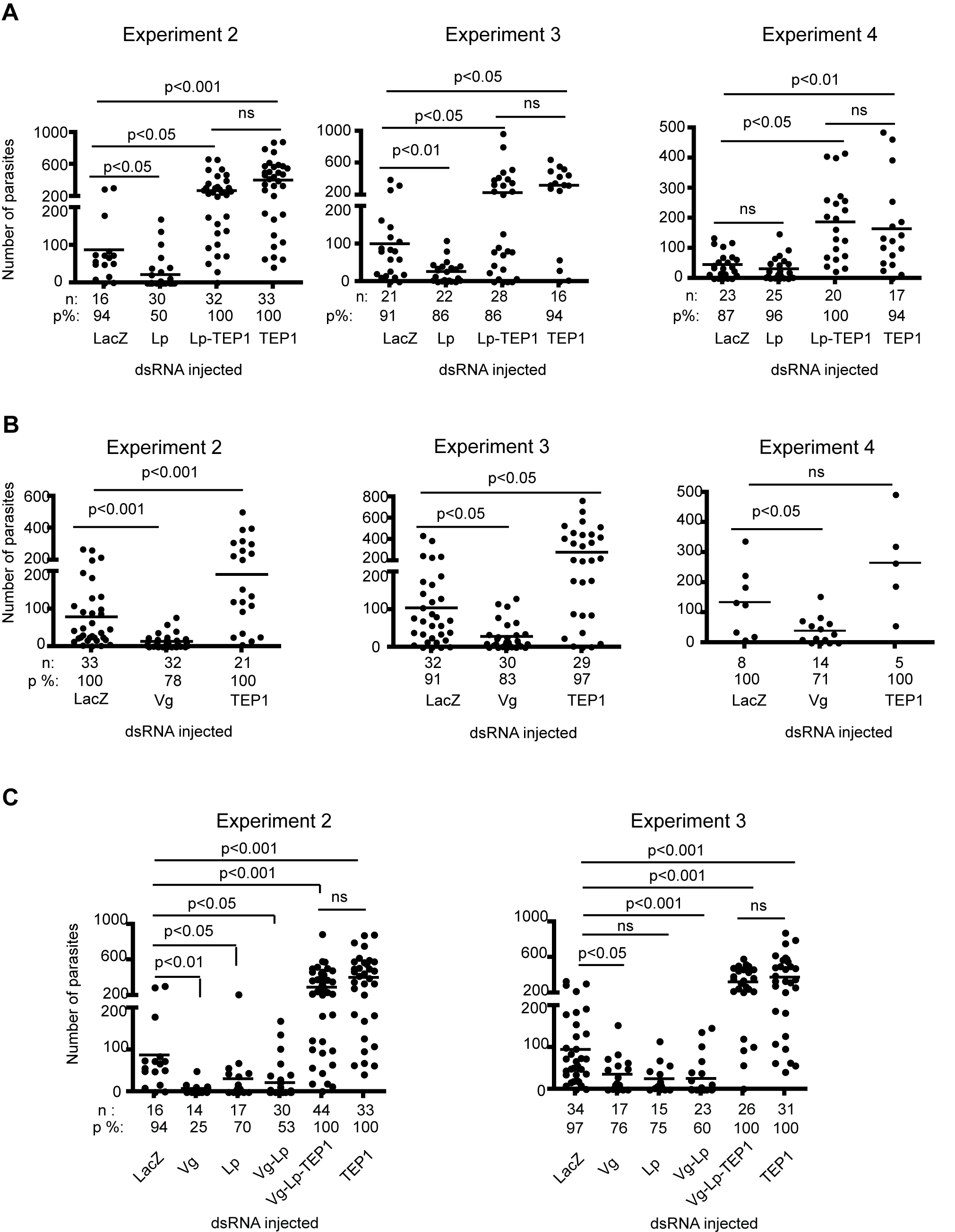

Supplement: Figure S3 — (A) Three additional repeats of the experiment shown in Figure 1A. (See Figure 1A for legend.) (B) Three additional repeats of the experiment shown in Figure 1B. (See Figure 1B for legend.) (C) Two additional repeats of the experiment shown in Figure 2A. (See Figure 2A for legend.) (0.35 MB TIF) [file pbio.1000434.s003.tif]
