# Supplementary material for: The Major Yolk Protein Vitellogenin Interferes with the Anti-Plasmodium Response in the Malaria Mosquito Anopheles gambiae
Source: PLoS Biol. 2010 Jul 20;8(7):e1000434. doi: 10.1371/journal.pbio.1000434 (PMC2907290; doi:10.1371/journal.pbio.1000434)
Supplement: Text S1 — The supplemental text describes lipophorin particle purification from adult mosquitoes by potassium bromide gradient fractionation or immuno-precipitation and a search for immune factors that co-purify with lipophorin. (0.10 MB DOC) [file pbio.1000434.s005.doc]

**Supplemental Text**

**Prophenoloxidase, but not the components of the TEP1 pathway, co-fractionates with lipophorin particles**

The fact that physiological levels of Lp (and Vg) seemed to dampen TEP1-dependent *Plasmodium* killing prompted us to examine whether TEP1 activity is modulated by a physical association of TEP1 with Lp. This idea was supported by a body of literature connecting: (i) vertebrate HDL and some effectors of innate immunity [1,2], including complement factor C3 [3,4], which belongs to the same family as TEP1; (ii) Lp and immune reactions in insects [5-8]; and (iii) the newly characterized role of Lp particles as vehicles for morphogen and glycosylphosphatidylinositol-linked proteins in *Drosophila* imaginal discs [9]. Lipidic particles from mosquito adults were purified using potassium bromide (KBr) gradient fractionation. Coomassie staining of SDS-PAGE gels showed that the two subunits of lipophorin accounted for most of the protein detectable in the top fraction of the KBr gradient (Suppl. Fig. 1A). To determine whether some TEP1 co-fractionates with Lp, we performed immunoblotting analysis with anti-TEP1 antibodies to probe the gradient fractions. To maximize detection, we concentrated the Lp-containing fractions 10 times compared to the protein-rich, bottom fractions. We could not detect TEP1 (except, occasionally, faint traces thereof) in the Lp-containing gradient fractions at various time points before or after infection, suggesting that this major *Plasmodium*-killing molecule is not significantly bound to Lp particles under these experimental conditions (Suppl. Fig. 1A). Interestingly, a prophenoloxidase (PPO, mediating melanotic encapsulation during insect defense) recognized by an anti-PPO2 antibody was present in the Lp-containing fractions (Suppl. Fig. 1B), indicating that some facets of mosquito immunity may involve an interaction between Lp and PPO. Indeed, such an association has been reported in studies of bacterial lipopolysaccharide aggregation in Lepidoptera [6] and of hemolymph clotting in A. gambiae larvae, in which PPO3 seems to assist Lp particles coalescence into sheet-like structures during clot formation [7]. PPO may interact directly with Lp, or co-fractionate in the low density part of the gradient via its association with uncharacterized lipidic compounds. Although PPO mediates melanization of *Plasmodium* ookinetes in some mosquito strains [10], previous work in our laboratory [11,12] established that this process occurs after parasite death and, therefore, probably does not play a part in parasite killing in *A. gambiae*.

We worried that the absence of TEP1 in lipophorin-containing fractions might be due to disruption of some molecular interactions at the high salt concentrations (3M KBr) used for gradient fractionation. Therefore, we sought to purify Lp particles by immunoprecipitation in physiological buffers. To this end, Lp purified by KBr fractionation was used to immunize mice to generate monoclonal antibodies. Eight antibodies were recovered, all of which recognized either the large or the small subunit of lipophorin. We selected an antibody directed against the small subunit (ApoLpII) that proved efficient for Lp immunoprecipitation in detergent-free buffer. Immunoblotting analysis of the immunoprecipitated Lp particles confirmed the absence of TEP1 (Suppl. Fig. 1C), though a small amount of the C-terminal TEP1 cleavage product was often pulled down non-specifically by Sepharose beads regardless of the presence of anti-Lp antibodies. Likewise, while control Sepharose beads in combination with non-specific antibodies did not pull down any Lp, they non-specifically pulled down substantial amounts of PPO and Vg in detergent-free buffer (data not shown). This prevented us from obtaining an independent confirmation for the co-purification of Lp and PPO observed in low-density KBr fractions.

We extended the analysis to two known components of the TEP1 protein complex, LRIM1 and APL1 [13,14], and asked whether Lp could modulate TEP1 function by sequestering these proteins. To this end, we probed the blots using anti-LRIM1 and anti-APL1 antibodies. No signal was detected with either antibody in Lp-containing fractions, suggesting that these proteins do not associate with Lp purified either by KBr fractionation or by immunoprecipitation (Suppl. Fig. 1A and data not shown). Thus, although Lp co-purifies with a fraction of PPO and perhaps with other, yet unknown, immune factors, its adverse effect on TEP1 activity is apparently not explained by a physical interaction between Lp and the known components of the TEP1 pathway.

**References**

1. Raper J, Fung R, Ghiso J, Nussenzweig V, Tomlinson S (1999) Characterization of a novel trypanosome lytic factor from human serum. Infect Immun 67: 1910-1916.

2. Pays E, Vanhollebeke B (2009) Human innate immunity against African trypanosomes. Curr Opin Immunol 21: 493-498.

3. Vaisar T, Pennathur S, Green PS, Gharib SA, Hoofnagle AN, et al. (2007) Shotgun proteomics implicates protease inhibition and complement activation in the antiinflammatory properties of HDL. J Clin Invest 117: 746-756.

4. Lange S, Dodds AW, Gudmundsdóttir S, Bambir SH, Magnadóttir B (2005) The ontogenic transcription of complement component C3 and Apolipoprotein A-I tRNA in Atlantic cod (Gadus morhua L.)--a role in development and homeostasis? Dev Comp Immunol 29: 1065-1077.

5. Kato Y, Motoi Y, Taniai K, Kadono-Okuda K, Yamamoto M, et al. (1994) Lipopolysaccharide-lipophorin complex formation in insect hemolymph: a common pathway of lipopolysaccharide detoxification both in insects and in mammals. Insect Biochem Mol Biol 24: 547-555.

6. Rahman MM, Ma G, Roberts HL, Schmidt O (2006) Cell-free immune reactions in insects. J Insect Physiol 52: 754-762.

7. Agianian B, Lesch C, Loseva O, Dushay MS (2007) Preliminary characterization of hemolymph coagulation in Anopheles gambiae larvae. Dev Comp Immunol 31: 879-888.

8. Whitten MM, Tew IF, Lee BL, Ratcliffe NA (2004) A novel role for an insect apolipoprotein (apolipophorin III) in beta-1,3-glucan pattern recognition and cellular encapsulation reactions. J Immunol 2004 Feb 15;172(4):2177-85 172: 2177-2185.

9. Panáková D, Sprong H, Marois E, Thiele C, Eaton S (2005) Lipoprotein particles are required for Hedgehog and Wingless signalling. Nature 435: 58-65.

10. Volz J, Muller HM, Zdanowicz A, Kafatos FC, Osta MA (2006) A genetic module regulates the melanization response of *Anopheles* to *Plasmodium*. Cell Microbiol 8: 1392-1405.

11. Blandin S, Levashina EA (2004) Mosquito immune responses against malaria parasites. Curr Opin Immunol 16: 16-20.

12. Shiao SH, Whitten MM, Zachary D, Hoffmann JA, Levashina EA (2006) *Fz2* and *cdc42* mediate melanization and actin polymerization but are dispensable for *Plasmodium* killing in the mosquito midgut. PLoS Pathog 2: e133.

13. Fraiture M, Baxter RH, Steinert S, Chelliah Y, Frolet C, et al. (2009) Two mosquito LRR proteins function as complement control factors in the TEP1-mediated killing of Plasmodium. Cell Host Microbe 5: 273-284.

14. Povelones M, Waterhouse RM, Kafatos FC, Christophides GK (2009) Leucine-rich repeat protein complex activates mosquito complement in defense against Plasmodium parasites. Science 324: 258-261.
